# Insights into the Light Response of *Skeletonema marinoi*: Involvement of Ovothiol

**DOI:** 10.3390/md18090477

**Published:** 2020-09-20

**Authors:** Alfonsina Milito, Ida Orefice, Arianna Smerilli, Immacolata Castellano, Alessandra Napolitano, Christophe Brunet, Anna Palumbo

**Affiliations:** 1 Department of Biology and Evolution of Marine Organisms, Stazione Zoologica Anton Dohrn, Villa Comunale, 80121 Napoli, Italy; immacolata.castellano@szn.it; 2 Department of Molecular Genetics, Centre for Research in Agricultural Genomics, Cerdanyola, 08193 Barcelona, Spain; 3 Department of Marine Biotechnology, Stazione Zoologica Anton Dohrn, Villa Comunale, 80121 Napoli, Italy; ida.orefice@szn.it (I.O.); arianna.smerilli@szn.it (A.S.); christophe.brunet@szn.it (C.B.); 4 Department of Chemical Sciences, University of Naples “Federico II”, 80126 Naples, Italy; alessandra.napolitano@unina.it

**Keywords:** algae, antioxidant, diatoms, light, nitric oxide, ovothiol, oxidative stress

## Abstract

Diatoms are one of the most widespread groups of microalgae on Earth. They possess extraordinary metabolic capabilities, including a great ability to adapt to different light conditions. Recently, we have discovered that the diatom *Skeletonema marinoi* produces the natural antioxidant ovothiol B, until then identified only in clams. In this study, we investigated the light-dependent modulation of ovothiol biosynthesis in *S. marinoi*. Diatoms were exposed to different light conditions, ranging from prolonged darkness to low or high light, also differing in the velocity of intensity increase (sinusoidal *versus* square-wave distribution). The expression of the gene encoding the key ovothiol biosynthetic enzyme, *ovoA*, was upregulated by high sinusoidal light mimicking natural conditions. Under this situation higher levels of reactive oxygen species and nitric oxide as well as ovothiol and glutathione increase were detected. No ovoA modulation was observed under prolonged darkness nor low sinusoidal light. Unnatural conditions such as continuous square-wave light induced a very high oxidative stress leading to a drop in cell growth, without enhancing ovoA gene expression. Only one of the inducible forms of nitric oxide synthase, *nos2*, was upregulated by light with consequent production of NO under sinusoidal light and darkness conditions. Our data suggest that ovothiol biosynthesis is triggered by a combined light stress caused by natural distribution and increased photon flux density, with no influence from the daily light dose. These results open new perspectives for the biotechnological production of ovothiols, which are receiving a great interest for their biological activities in human model systems.

## 1. Introduction

Diatoms represent one of the most widespread and diversified groups of unicellular photosynthetic eukaryotes, widely distributed in all aquatic environments. Due to their relevant abundance in marine ecosystems, they contribute to one-fifth of the photosynthesis carried out on Earth [1] and play a crucial role in biogeochemical cycles [2]. Moreover, diatoms can provide a rich source of bioactive products, including carotenoids, vitamins and polyunsaturated fatty acids [3,4,5], thus representing promising “biofactories” for biotechnological applications [6,7].

Due to the complex evolutionary history and multiple horizontal gene transfer events from bacteria and viruses [8,9], diatoms possess unique metabolic capabilities, allowing them to adapt to a plethora of ecological niches and to efficiently cope with the environmental forcing variability [10,11,12,13,14]. Among such environmental variables, light is a key factor influencing diatom growth and physiology. Indeed, diatoms are passively transported along the water column, and thus are exposed to very fast changes in light intensity and spectral composition. In a short time frame, the same species can switch from darkness or a very low light intensity environment—with dominance of blue and/or green as well as absence of red wavelength—to very high light intensity characterized by the full range of visible wavelengths (400–700 nm) [14]. Diatoms evolved an efficient ability to adapt to different light conditions by modulating the levels of photosynthetic and photoprotective pigments, as well as of antioxidant compounds, especially carotenoids [15,16,17,18,19]. Among carotenoids, pigments forming the xanthophyll cycle (XC) are responsible for most of the short-term photoprotection, including the non-photochemical quenching of chlorophyll α fluorescence (NPQ) [18,20,21,22]. High light intensity, or more generally light induced stress, also promotes the synthesis of other antioxidants, such as ascorbic and phenolic acid, as well as the activation of antioxidant enzymes [18,19].

Recently, we have discovered that the coastal centric diatom *Skeletonema marinoi*, grown under moderate light condition, produces micromolar concentrations of ovothiol B [23], hitherto identified only in clams [24].

Ovothiol B belongs to the π-methyl-5-thiohistidines class (ovothiols), considered powerful antioxidants and mainly found in marine invertebrates, proteobacteria and protists, e.g., microalgae [25]. Due to the aromaticity of the imidazole ring, ovothiols possess a highly acidic thiol group (pKa = 1.4) compared to other cellular thiols, such as glutathione (pKa = 8.75) [26,27,28]. This chemical feature is related to the ability of ovothiols to act as efficient scavengers of radicals and peroxides [29]. Ovothiols have been receiving great interest for their biological activities in *in vitro* and *in vivo* human model systems. Indeed, they exhibited pleiotropic beneficial properties, revealing antifibrotic activities in a murine model of liver fibrosis [30,31], antiproliferative action in human cancer cell lines [32,33,34] and anti-inflammatory effects in endothelial cells derived from women affected by gestational diabetes [35]. Ovothiols are synthesized *in vivo* by two key enzymes, the sulfoxide synthase OvoA and the β-lyase OvoB [36,37], in three different forms, A, B and C, differing in the methylation state of the α-amino group. Ovothiol B is a monomethylated form, while ovothiol A lacks this methyl and ovothiol C possesses two methyl groups [25].

Only few studies performed on microalgae have highlighted some connections between ovothiol biosynthesis and light-dependent processes. Indeed, ovothiol was reported to be a redox regulator, controlling the activity of the chloroplast-coupling factor in *Dunaliella salina* [38]. Moreover, different ovoA transcripts were identified in *Euglena gracilis* under dark or sunlight conditions [39].

The present study is intended to explore more systematically the possible role of ovothiol in the light-dependent response of the diatom *S. marinoi* and to understand if this poorly known antioxidant could contribute to defend diatoms from light stress. To this aim, *S. marinoi* cells were exposed to different light regimes, varying in photon flux density (PFD), velocity of intensity increase (sinusoidal *versus* square-wave distribution) and light:dark photoperiod cycle. We tested the hypothesis that changes in light conditions could affect the expression of the gene *ovoA* encoding the key ovothiol biosynthetic enzyme. Moreover, to investigate whether ovothiol biosynthesis may be part of the cellular antioxidant response, we monitored the cellular stress status by measuring the concentration of reactive oxygen species (ROS) and nitric oxide (NO), important messengers of stress response [40,41,42,43], as well as the expression of the enzyme responsible for NO biosynthesis, e.g., NO synthase, recently identified in diatoms [44]. We also tested the possibility that light-induced upregulation of *ovoA* gene expression could result in an increased ovothiol production, activating the complete biosynthetic machinery necessary for its biosynthesis. The outcomes of the present work open new perspectives on the possible exploitation of diatoms for a large-scale production of this compound. Indeed, diatoms’ biomass enriched with antioxidant molecules (ovothiol, carotenoids, polyphenols, etc.) may represent an ecofriendly solution for biotechnological purposes, especially using light as a manipulating factor [45].

## 2. Results

### 2.1. Molecular Response to High Light Conditions

We evaluated the molecular response of the diatom *Skeletonema marinoi* under a moderate light condition, used as a non-stressful control (low sinusoidal light, having midday light intensity peak at 150 µmol photons s^−1^ m^−2^, Sin150), as well as under high light stressful conditions, including high sinusoidal light peaking at 600 µmol photons s^−1^ m^−2^ (Sin600), high square-wave light peaking at 300 (Square300) and 600 µmol photons s^−1^ m^−2^ (Square600). These light conditions vary in both light intensity distribution over time (sinusoidal *vs*. square-wave), in the midday light intensity peak (150 *vs.* 300 *vs.* 600 µmol photons s^−1^ m^−2^) and in the daily light dose (3.6 mol photons m^−2^ d^−1^ for Sin150, 14.4 mol photons m^−2^ d^−1^ for Sin600 and Square300, 28.8 mol photons m^−2^ d^−1^ for Square600). 

Under the different experimental conditions, we assessed the expression of *ovoA* and *nos* genes to highlight their eventual light modulation at different time points. Samples were taken at 0, 6 and 24 h under all conditions, while additional samples were included in the high stressful light conditions. An earlier time point (2 h) was taken under Sin600 and two additional samplings (0.2 and 2 h) were carried out under Square300 and Square600 conditions, to evaluate the early response under high light stress. In *S. marinoi* two nos transcripts have been previously identified but no data are available on their functional significance [44]. Interestingly, our *in silico* analysis showed that both *S. marinoi* Nos protein sequences lack the inhibitory loop (Supplementary Figure S1), which is a feature of inducible Nos, making it Ca^2+^-independent [46]. To detect the redox status of the cells, in all experimental conditions we also measured the levels of reactive oxygen species (ROS) and nitric oxide (NO) through biochemical assays.

**Figure 1:**
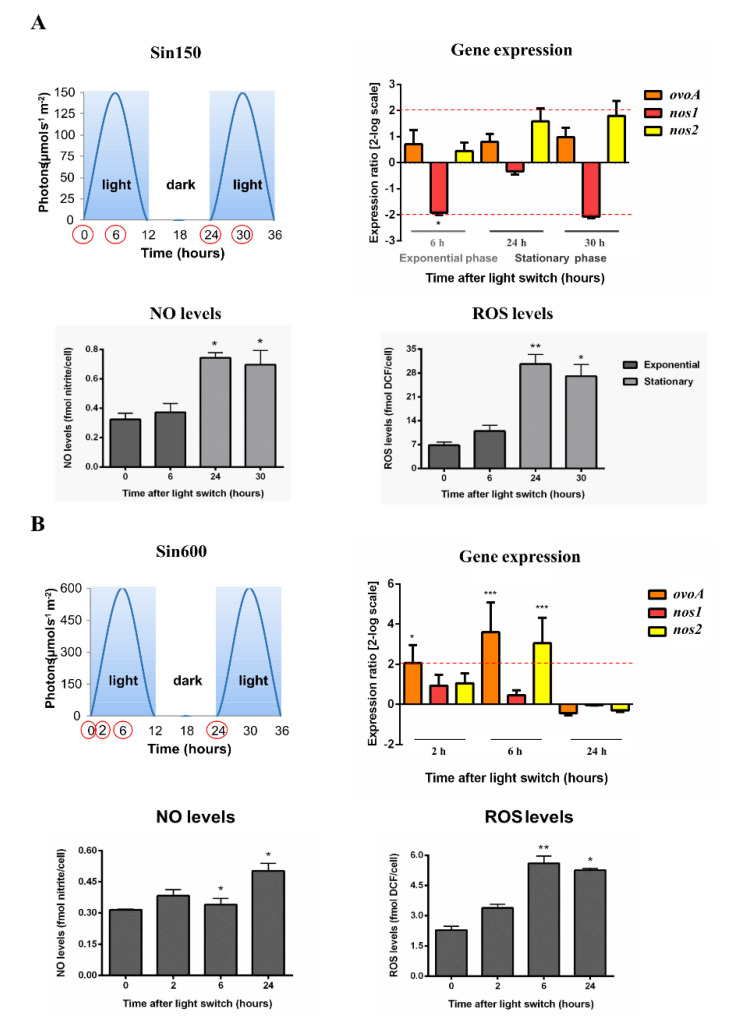
*S. marinoi* under control and high sinusoidal light conditions. Scatter plot representing light condition, gene expression data, NO and ROS levels were reported. (**A**) Control condition with midday peak at 150 µmol photons s^−1^ m^−2^ (low sinusoidal light, Sin150); (**B**) High sinusoidal light with midday peak at 600 µmol photons s^−1^ m^−2^ (Sin600). The light:dark photoperiod cycle was 12 h:12 h. Sampling times were highlighted in the light scatter plot by red circles. Fold gene expression data were analyzed by the pairwise fixed reallocation randomization test by REST and are here reported as 2-log scale mean ± standard error and. NO and ROS data were analyzed by Kruskal–Wallis with a Dunn’s post hoc test and are reported as mean ± standard deviation. * *p* < 0.05, ** *p* < 0.01 and *** *p* < 0.001 represent significance compared to 0 time.

Under the control light condition (Sin150), no modulation of *ovoA* expression was observed at any experimental time both in exponential and in stationary growth phases (Figure 1A). The same was observed for *nos1* and *nos2*, except for a *nos1* down-regulation at the midday light intensity peak in exponential growth phase (6 h), with a decreasing trend also at the midday peak in the stationary phase (30 h, Figure 1A). Both NO and ROS levels were higher in stationary compared to the exponential growth phase, while they did not vary during the day in the exponential growth phase, confirming the non-stressful nature of this control condition (Figure 1A). Cultures exposed to Sin600 showed an upregulation of *ovoA* gene expression after 2 and 6 h from the light switch (Figure 1B). Additionally, *nos2* upregulation was observed after 6 h, while no variation was observed for *nos1* (Figure 1B). Both NO and ROS levels increased at 6 h and 24 h from light switch to Sin600 (Figure 1B).

To investigate whether the *ovoA* gene upregulation observed at 6 h of Sin600, compared to Sin150, resulted also in increased production of the molecule, we measured the concentration of ovothiol both under Sin600 and Sin150, at the midday light intensity peaks 600 and 150 µmol photons s^−1^ m^−2^, respectively. We also evaluated the content of the other major intracellular thiol glutathione. In agreement with the upregulation of *ovoA*, the cellular content of ovothiol B increased by two-fold from 50 µM under Sin150 to 110 µM under Sin600 (Figure 1A; Table 1). Similarly, glutathione also doubled its concentration from 1 mM under control condition (Sin150) to 2.3 mM under high sinusoidal light (Sin600; Table 1).

**Figure 2:**
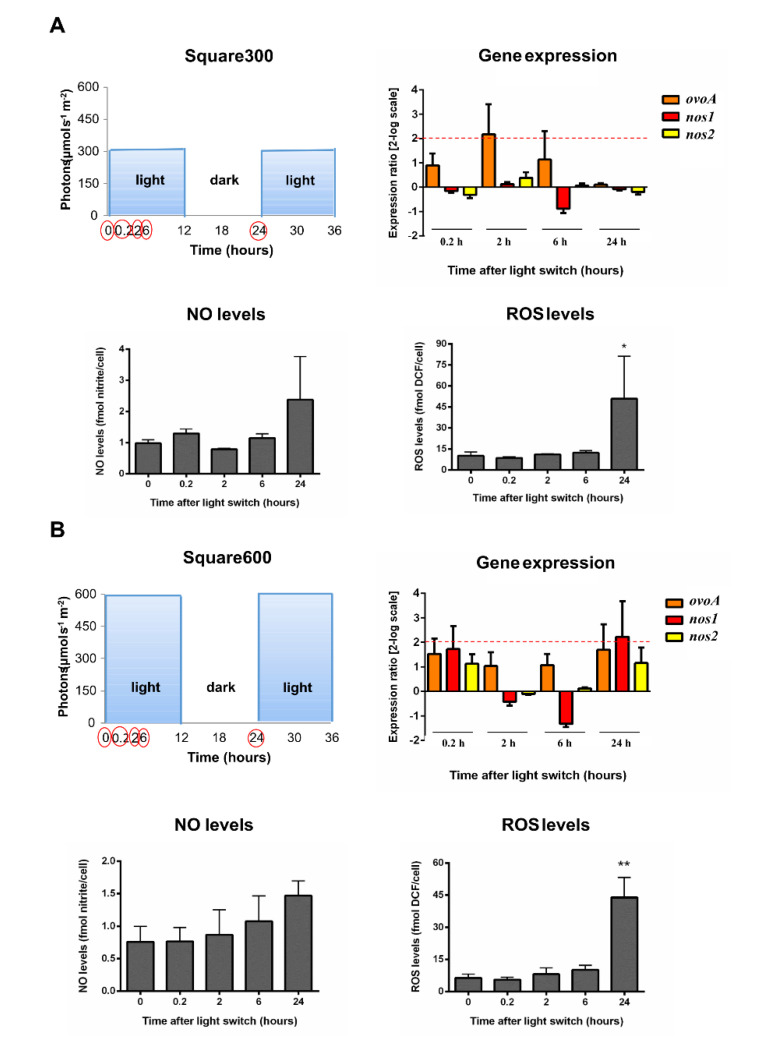
*S. marinoi* under high square-wave light conditions. Scatter plot representing light condition, gene expression data, NO and ROS levels were reported. (**A**) High square-wave light with midday peak at 300 µmol photons s^−1^ m^−2^ (Square300); (**B**) High square-wave light with midday peak at 600 µmol photons s^−1^ m^−2^ (Square600). The light:dark photoperiod cycle was 12 h:12 h. Sampling times were highlighted in the light scatter plot by red circles. Fold gene expression data were analyzed by the pairwise fixed reallocation randomization test by REST and are here reported as 2-log scale mean ± standard error and. NO and ROS data were analyzed by Kruskal–Wallis with a Dunn’s post hoc test and are reported as mean ± standard deviation. * *p* < 0.05 and ** *p* < 0.01 represent significance compared to 0 time.

Cells moved to Square300 did not show any significant modulation in the expression of *ovoA* nor of *nos*, neither at a very early time point (0.2 h). The concentrations of NO and ROS did not increase, with the exception of a significant ROS overproduction at 24 h, following the night phase (Figure 2A). Similarly, no gene modulation was observed in the Square600 condition, at any experimental time, with ROS significantly increasing at 24 h (Figure 2B).

Interestingly, cells under both square-wave lights (Square300 and Square600) displayed a very high oxidative stress at 24 h, following the night phase (ROS content about 45 fmol DCF/cell; Figure 2A,B), compared to cells under Sin600 (ROS content about 5 fmol DCF/cell; Figure 1B). Indeed, this latter condition, although being characterized by a high intensity of light, mimicks a natural climate, progressively increasing the light intensity until the midday peak. Conversely, under the square-wave distribution, cells are suddenly exposed to the maximal light intensity, not allowing the acclimation process, which occurs in natural conditions. The detrimental effect of the square-wave light conditions was also visible by the drop in cell growth following the stress received (24 h), differently to what was observed in cells exposed to Sin600 (Figure S2 and Table S1).

### 2.2. Molecular Response to Prolonged Darkness and Low Light Conditions

In a next step, we assessed the molecular response to prolonged darkness (0 h:24 h light:dark photoperiod cycle) and very low light conditions (midday light intensity peak at 10 µmol photons s^−1^ m^−2^), i.e., Sin10 and Square10, varying in both light:dark photoperiod cycles (12 h:12 h and 24 h:0 h, respectively) and in light distribution over time (sinusoidal *vs.* square-wave, respectively). In these conditions, samplings were carried out at dawn and midday peak of two consecutive days (0, 6, 24 and 30 h) to follow two consecutive light:dark photoperiod cycles.

**Figure 3:**
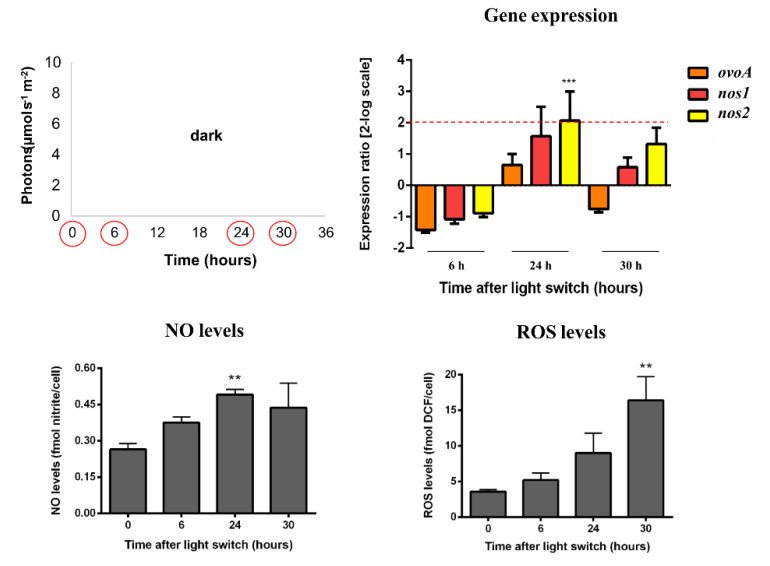
*S. marinoi* under dark condition. Scatter plot representing the dark condition, gene expression data, NO and ROS levels were reported. Dark was kept constant for all the experiment (light:dark photoperiod cycle 0 h:24 h). Sampling times were highlighted in the scatter plot by red circles. Fold gene expression data were analyzed by the pairwise fixed reallocation randomization test by REST and are here reported as 2-log scale mean ± standard error and. NO and ROS data were analyzed by Kruskal–Wallis with a Dunn’s post hoc test and are reported as mean ± standard deviation. ** *p* < 0.01 and *** *p* < 0.001 represent significance compared to 0 time.

Cells exposed to a continuous dark condition showed a significant *nos2* upregulation after 24 h without any *ovoA* modulation (Figure 3). NO levels increased at 24 h, while the concentration of ROS was upregulated at 30 h (Figure 3).

**Figure 4:**
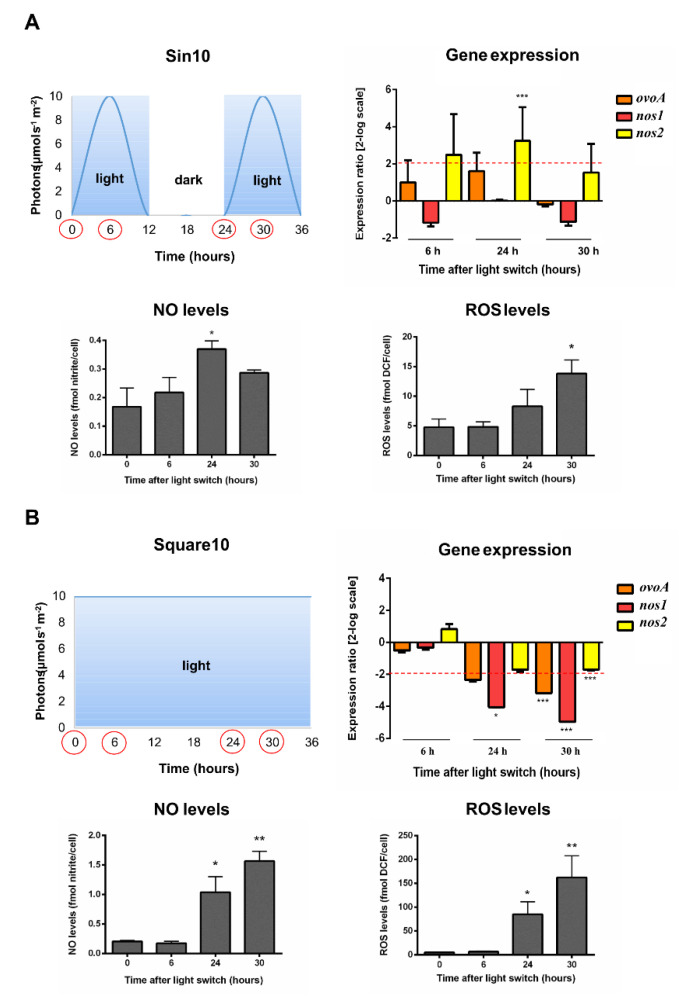
*S. marinoi* under very low sinusoidal and square-wave light conditions. Scatter plot representing light condition, gene expression data, NO and ROS levels were reported. (**A**) Very low sinusoidal light with a midday peak at 10 µmol photons s^−1^ m^−2^ (Sin10). The light:dark photoperiod cycle was 12 h:12 h; (**B**) Very low square-wave light was kept constant for all the experiment at 10 µmol photons s^−1^ m^−2^ (Square 10). The light:dark photoperiod cycle was 24 h:0 h. Sampling times were highlighted in the light scatter plot by red circles. Fold gene expression data were analyzed by the pairwise fixed reallocation randomization test by REST and are here reported as 2-log scale mean ± standard error and. NO and ROS data were analyzed by Kruskal–Wallis with a Dunn’s post hoc test and are reported as mean ± standard deviation. * *p* < 0.05, ** *p* < 0.01 and *** *p* < 0.001 represent significance compared to time 0.

Cells shifted to Sin10 did not modulate *ovoA* nor *nos1* gene expression, while *nos2* was upregulated after 24 h from the light switch (Figure 4A). NO and ROS variations under Sin10 condition resembled the pattern obtained under prolonged darkness. Indeed, levels increased after 24 h and 30 h, for NO and ROS respectively (Figure 4A).

Under continuous Square10 condition, *nos1* was downregulated after 24 h from the light switch, while all the target genes were significantly downregulated at 30 h. In parallel, NO and ROS increased levels were observed at the same times, with ROS reaching very high concentrations (about 100–150 fmol DCF/cell, Figure 4B).

Among these three conditions, Square10 resulted to be the most stressful for the cells, as also highlighted by the drop in cell growth observed at 24 h (Supplementary Table S1).

Pairwise Pearson correlation analyses were performed between the different variables in three clusters of light conditions: low light (Sin150, Sin10 and Square10), high light (Sin600, Square300 and Square600) and dark. In the high light cluster, *ovoA* was correlated to *nos2*, and ROS levels were positively correlated to NO and *nos1* (Table 2). Similarly, under low light NO was correlated with ROS while *ovoA* with both *nos1* and *nos2*, and *nos1* with *nos2* (Table 2). In the dark condition, only NO-*ovoA* and ROS-*nos1* pairs were correlated (Table 2). These results displayed the different pattern and potential interactions between these variables shaped by the light quantity harvested by cells.

## 3. Discussion

Ovothiols are natural sulfur compounds mainly occurring in marine organisms and in the microbial world, in three differentially methylated forms: A, B and C [25]. The biological role of these molecules has often been linked to their peculiar antioxidant properties [26,27] mediating defense from oxidative stress during fertilization and development in the sea urchin [47,48], from environmental stressors in fish eye lenses [49], from macrophage-triggered stress in pathogenic parasites during infection [50] and from light-dependent stress in microalgae [38,39]. In addition, ovothiols have been recently suggested to protect the sea anemone from the stress induced by UV radiation [51] and mussels from environmental pollutants during spawning [52]. However, biological functions not strictly related to oxidative stress have also been discovered. For example, ovothiols have been reported to induce egg release in marine polychaetes during sexual reproduction [53]. Interestingly, ovothiols might play important roles also in animals that, although lacking the biosynthetic pathway, might acquire this bioactive compound from external sources [49,54,55].

We recently described the occurrence and distribution of OvoA in diatoms and we identified ovothiol B as the ovothiol form produced by the coastal centric diatom *Skeletonema marinoi* at micromolar concentrations, when grown under moderate light condition [23]. Our hypothesis is that ovothiol biosynthesis could have been evolved and conserved in diatoms to help them to defend from the oxidative stress enhanced by high light, thus contributing to the ecological success of these photosynthetic protists. Following this assumption, we investigated the light-dependent regulation of ovothiol B biosynthesis in *S. marinoi*, mainly examining the expression of the gene *ovoA* encoding the key ovothiol biosynthetic enzyme.

The experimental strategy adopted in this study involved the cultivation of *S. marinoi* cells under different stressful light conditions, following a preacclimation period under moderate light. For each condition, samples were collected at different time points and were analyzed for gene expression of *ovoA* as well as for intracellular concentration of reactive oxygen species (ROS) and nitric oxide (NO), considered key mediators of stress response [11,40,41,42,43,56]. To integrate data on NO production and to investigate a possible involvement of the arginine-dependent NO biosynthesis in response to stressful light conditions, we also followed the gene expression of NO synthases in *S. marinoi*, which we named *nos1* and *nos2*. Among all the conditions tested, the high sinusoidal light (Sin600) resulted to be the most efficient in inducing an increased expression of *ovoA*. Indeed, *ovoA* is upregulated after only 2 and 6 h from the light switch with a significant increase in NO and ROS content at 6 h, as well as ovothiol production, which doubles its concentration compared to the control condition. Under this light, also *nos2* is upregulated after 6 h from a light switch together with increased NO levels already at 6 h and maintained high until 24 h, while *nos1* is not modulated at any experimental time. This may indicate a different role for these two nos transcripts. The *in silico* analysis of the Nos1/2 protein primary structure points out an inducible nature of the two Nos isoforms, but our data suggest that only Nos2 responds to high light, with a concomitant increase of NO. This issue is quite interesting and would deserve further investigation to understand which factors may be involved in Nos1 induction.

The finding that *ovoA* is not modulated in cells under square-wave light conditions suggests that the exposure to fast increases in photon flux density (PFD) does not allow the cells to efficiently modulate their metabolism, as also evident from the very high oxidative stress observed in cells at 24 h from the light switch to Square300 and Square600. Indeed, these unnatural conditions likely compromise the physiological defense mechanisms, finally leading to cell death, as indicated by the observed drop in cell growth. Even at a very early time (0.2 h) the cells do not exhibit any response in terms of *ovoA* modulation and other measured variables. Interestingly, our data are in line with a recent study evaluating the overall response of *S. marinoi* to different light regimes in terms of activated photoprotective and antioxidant systems, including ascorbic acid, one of the most abundant intracellular antioxidants, carotenoids and phenolic compounds, which significantly increase under the Sin600 condition [19]. Thus, the finding that both ovothiol and glutathione double their content in Sin600 condition may indicate that this light regime is able to modulate the diatom metabolism such that all the triggered photoprotective and antioxidant systems may synergically contribute to the defense from the high light stress. Indeed, the increased ovothiol production under the Sin600 condition suggests that this condition not only induces OvoA activity but also all the enzymatic toolkit necessary for ovothiol B biosynthesis, including the lyase OvoB and the still uncharacterized methyltransferase [36,37,55]. Moreover, the uncommon antioxidant properties of ovothiols compared to other thiols, including glutathione [26,27,28], can partially explain the observed difference in concentration of these two thiols in *S. marinoi*, suggesting that very low levels of ovothiol could be enough to exploit its antioxidant function.

The lack of *ovoA* modulation in Square300 highlights that the daily light dose is not a trigger for the increase of *ovoA* expression, since this condition is characterized by the same daily light dose as Sin600 (around 14.4 mol photons m^−2^ for both Sin600 and Square300), which is conversely able to stimulate ovothiol biosynthesis. Moreover, ovothiol biosynthesis does not react to fast increases of PFD, provided with Square600 condition, but instead to a gradual PFD increase under Sin600 condition.

These results highlight the phenotypic plasticity of *S. marinoi* when submitted to different light conditions, with ovothiol involvement into the photoresponse when cells cope with natural high light condition (sinusoidal), while it does not take part to the response of cells to unnatural high light condition. This is a quite interesting issue being that phenotypic plasticity, defined as the ability of an organism’s genotype to display different phenotypes in response to the environmental variability, is a key process explaining microalgal adaptability to natural conditions [57,58,59]. In particular, microalgae are adapted to a natural light sinusoidal distribution during the day, allowing them to efficiently cope with light variations, e.g., high light environment, through the activation of different kinds of complementary photoresponses, from the fast non-photochemical quenching or xanthophyll cycle operation to changes in carotenoids levels or in antioxidant network activation. When cells are submitted to unnatural light variations (e.g., square-wave distribution or very fast light increase), they undergo physiological/biochemical stress responses, which are different in amplitude and time succession sequence compared to responses to natural light conditions [16,17,18,19,60].

The results of our study let us hypothesize that ovothiol synthesis/activation is among the players into the natural physiological response of microalgae to high light, but seems not to be a way for cells to cope with highly stressful (unnatural) light conditions. This might be due to the high-energy cost for cells to synthesize ovothiol, or, more likely, to the lack of biological intracellular signal for the activation of ovothiol biosynthesis.

Yet, the lack of *ovoA* modulation during the stationary growth phase reinforces the finding that the transcriptional level of *ovoA* responds to light and is not modulated by other physiological stressful processes, such as senescence.

The results on *ovoA* modulation under darkness and very low light conditions confirm the key role of light in enhancing ovothiol biosynthesis. Indeed, while there is basically no *ovoA* modulation under prolonged darkness and Sin10 condition (12 h:12 h light:dark), cells grown under continuous Square10 (24 h:0 h light:dark) display a downregulation of the genes *ovoA* and *nos1*/*2* over time, reaching a high significance at 30 h from the light switch, together with an increase of NO and ROS levels, and a drastic decrease in cell growth rate. This may be due to the extremely unnatural character of this condition. By contrast, diatoms can tolerate prolonged darkness and very low sinusoidal light, since they naturally experience these conditions when they sink at the limit or below the photic zone. These results may indicate that the absence of *ovoA* upregulation under darkness and low light conditions leads to very low levels of ovothiol, which are presumably not enough to counteract the enhanced oxidative stress. Interestingly also other antioxidants and protective systems do not increase in dark and low light conditions, thus underlining a crucial role of light to induce the production of photoprotective and antioxidant systems, excluding an internal circadian clock [19].

Overall, the conditions mostly affecting the variables here measured are Sin600 and Square10. Indeed, while a too high intensity light could damage the photosynthetic apparatus of diatoms, also a prolonged light, even though at very low light intensity, could cause a chronic accumulation of ROS and lead to cell death, as observed. However, the upregulation of antioxidant systems, including ovothiol biosynthesis, by high sinusoidal light allows the cells to better tolerate the stress, while cells experiencing a low light (or dark) stress are not able to efficiently respond due to the absence of light modulation of such defense systems.

The present work might provide the basis for a possible eco-sustainable production of ovothiols by diatoms, to be used as “biofactories” for biotechnological purposes through the light modulation of their growth, metabolism and physiology [3,7,19,61,62,63]. Indeed, despite the increasing interest in the pharmaceutical potential of this class of molecules endowed with antiproliferative, anti-inflammatory and antifibrotic properties [30,31,32,33,34,35], studies regarding their bioactivities are still limited by the small amounts of ovothiol available for applied research. Currently, they can be extracted by sea urchin eggs, thus obtaining 2.5 mg of pure ovothiol A from 10 g of wet material [25,32]. However, sea urchins cannot provide sufficient amounts of ovothiols for extensive studies, also considering the necessity to preserve natural populations at sea. On the other hand, the light-induced upregulation of ovothiol formation by 2-fold might still be not enough to allow an efficient exploitation of such system for biotechnological purposes. Therefore, further studies will be necessary to find alternative and more efficient growth conditions, e.g., different nutrient availabilities, that might be able to increase ovothiol levels more efficiently, or genetic engineering of diatoms to optimize the ovothiol biosynthesis by gene manipulation.

## 4. Materials and Methods

### 4.1. Experimental Strategy and Sampling

The coastal centric diatom *Skeletonema marinoi* Sarno and Zingone (strain CCMP 2092) was used as the model species in this work for its high growth rate [16,64] and its use in aquaculture and biotechnology [18,19,65,66,67,68]. Additionally, the biology and photophysiology of this species are well known [16,63]. The strain (CCMP 2092) used in this work was collected from surface waters of the Northern Adriatic Sea, where this diatom provides the major contribution to the late winter blooms [69]. *S. marinoi* cultures were carried out in 4.5 L glass flasks under water movement, provided by an aquarium wave maker pump (Sunsun, JVP-110) at 20 °C, containing seawater previously prefiltered through a 0.7 μm GF/F glass-fiber filter (WhatmanTM) autoclaved and enriched with F/2 medium nutrients [70] with some modifications. In particular, to ensure enough nutrient supply, the content of essential nutrients (phosphate, dissolved silica, trace metals and vitamins) in the culture medium was doubled [63]. Light was provided by a custom-built LED illumination system, able to modulate light intensity and spectral composition (Patent number: EP 13196793.7) [14]. Light intensity was measured inside each flask by using a laboratory PAR4π sensor (QSL 2101, Biospherical Instruments Inc., San Diego, CA, USA). The light spectral composition was kept constant during the preacclimation and all the experimental conditions were set up with a ratio blue:green:red = 50:40:10, usually found in the first layers of the photic zone at sea [14]. Preacclimation of cells was performed for a minimum of two weeks at a moderate light intensity, 12 h:12 h light:dark photoperiod, with a sinusoidal intensity distribution peaking at 150 µmol photons s^−1^ m^−2^ after 6 h from dawn (Sin150). Each experiment included three independent cultures and lasted 1–2 days, after the shift from the preacclimation condition to the experimental light condition. Both sinusoidal and square-wave light distributions were applied at different intensities in order to investigate the effect of different velocities of light increase on ovothiol biosynthesis [60]. Sinusoidal distribution is similar to the natural condition, with a succession of dawn, gradual light increase up to the midday intensity peak and gradual light decrease up to sunset. By contrast, the square-wave distribution—generally used in indoor algal cultivation systems—constitutes an unnatural condition, providing a fast increase of light intensity, kept constant for all the day phase (12 h) and then switched off for the night phase (12 h).

The seven experimental conditions were:

− Darkness (continuous absence of light; 0 h:24 h light:dark; daily light dose: 0 mol photons m^−2^);
− Very low sinusoidal light 10 (midday light intensity peak: 10 µmol photons s^−1^ m^−2^; 12 h:12 h light:dark; daily light dose: 0.24 mol photons m^−2^; Sin10);
− Very low square-wave light 10 (continuous light intensity: 10 µmol photons s^−1^ m^−2^; 24 h:0 h light:dark; daily light dose: 1 mol photons m^−2^; Square10);
− Low sinusoidal light 150 (midday light intensity peak: 150 µmol photons s^−1^ m^−2^; 12 h:12 h light:dark; daily light dose: 3.6 mol photons m^−2^; Sin150);
− High sinusoidal light 600 (midday light intensity peak: 600 µmol photons s^−1^ m^−2^; 12 h:12 h light:dark; daily light dose: 14.4 mol photons m^−2^; Sin600);
− High square-wave light 300 (continuous light intensity: 300 µmol photons s^−1^ m^−2^; 12 h:12 h light:dark; daily light dose: 14.4 mol photons m^−2^; Square300);
− High square-wave light 600 (continuous light intensity: 600 µmol photons s^−1^ m^−2^; 12 h:12 h light:dark; daily light dose: 28.8 mol photons m^−2^; Square600).

The Square300 condition provided the same daily light dose experienced by cells grown under Sin600, allowing us to compare square-wave and sinusoidal distributions of light, without any influence from different daily light doses [19]. All experimental conditions provided a 12 h:12 h light:dark photoperiod except for the conditions Square10 (very low intensity light was kept constant for all the duration of the experiment) and darkness (no “day phase” for all the duration of the experiment).

For each experimental condition, samplings were performed in the exponential growth phase, except for the control light condition (Sin150) in which samplings were done both in exponential and stationary growth phases.

In particular, for all the conditions samples were taken at predawn (time 0 h), at 6 h (midday peak) and 24 h (at the end of the night phase) from the light switch. Since high light is known to trigger rapid photoprotective processes in phytoplankton species [71] an additional sampling at 2 h was performed for Sin600, Square300 and Square600. In case of the square-wave light conditions (Square300 and Square600), in which cells experienced a very unnatural and fast increase of light intensity, samples were taken also at 0.2 h (12 min) to evaluate the short-term response. In case of very low sinusoidal and square-wave lights and darkness conditions, in which cells experienced different light:dark photoperiod cycles, samplings were done at 0 and 6 h for two consecutive days.

### 4.2. Cell Density

Cell density was monitored during the experiments to obtain growth curves. Briefly, 2 mL subsamples from each flask were collected and fixed with Lugol’s iodine solution (1.5% *v*/*v*). One milliliter of this solution was used to fill a Sedgewick Rafter counting cell chamber and cell counts were performed by using a Zeiss Axioskop 2 Plus light microscope (Carl Zeiss, Göttingen, Germany).

### 4.3. RNA Extraction, Reverse Transcription and Best Reference Genes Assessment

Diatom samples (50 mL) were collected at different times from dawn by centrifugation at 3200 rcf for 30 min at 4 °C. The final pellets were resuspended in 800 µL TRIZOL, frozen in liquid nitrogen and stored at −80 °C until use. The total RNA was extracted according to Barra et al., 2013 [41] and subjected to DNase treatment using DNase I recombinant, RNase-free (Roche, Basel, Switzerland), according to the manufacturer’s protocol. RNeasy MinElute Cleanup Kit (Qiagen, Venlo, The Netherlands) was used to purify and concentrate the total RNA, finally eluted in 20 μL RNase-free water. RNA samples were quantified by assessing the absorbance at 260 nm (ND-1000 Spectrophotometer; NanoDrop Technologies, Wilmington, DE, USA.) and then checked for integrity by agarose gel electrophoresis. Possible gDNA contamination was checked by PCR on RNA samples and agarose gel electrophoresis. From each RNA sample 1 μg was retrotranscribed in complementary DNA (cDNA) using the iScriptTM cDNA synthesis kit (Bio-Rad Laboratories, Hercules, CA, USA) and the T100 Thermal cycler (Bio-Rad Laboratories, Hercules, CA, USA), following the manufacturer’s instructions. In order to analyze the expression levels of target genes, five putative reference genes were analyzed by RT-qPCR to find the most stable genes in our conditions. The selected genes were histone 4 (*H4*), α- tubulin (*TUB A*), elongation factor 1α (*EF1α*), glyceraldehyde-3-phosphate dehydrogenase (*GAPDH*) and actin (*ACT*). For the amplification of putative reference genes, specific primers reported in the literature were used [72]. The best reference gene for each condition was identified by crossing results obtained with two different algorithms: BestKeeper [73] and NormFinder [74]. In particular, *H4* was used as a reference gene in the following conditions: Sin600, Square300 and darkness; *GADPH* was used for Square10; *ACT* for Square600 and *TUB A* for Sin10 and Sin150.

### 4.4. Reverse Transcription-Quantitative PCR (RT-qPCR) Experiments

For *ovoA*, the expression levels of the transcript SmOvoA_2388 containing all the canonical domains of metazoan OvoAs [23] were examined. For *nos* genes, the expression of two different transcripts previously reported in *S. marinoi* [44] was analyzed. We named the two *nos* genes: *nos1* and *nos2* (for details see Materials and Methods 4.8.). Specific primers were designed using Primer3 program V. 4.1.0 (primer3.ut.ee) considering the putative sequences reported in the *S. marinoi* transcriptome (MMETSP1039, http://datacommons.cyverse.org/browse/iplant/home/shared/imicrobe/camera/camera_mmetsp_ncgr). RT-qPCR was performed in a MicroAmp Optical 384-Well reaction plate (Applied Biosystems, Foster City, CA, USA) with optical adhesive covers in a Viia7 Real Time PCR System (Applied Biosystem, Foster City, CA, USA). The oligos used to amplify the target genes were reported in Table S2. Serial dilutions of cDNA and the obtained cycle (Ct) mean values were used to generate the standard curves in order to calculate primer reaction efficiency (E= 10^−1/slope^) and correlation factor R2 (Table S2). The RT-qPCR reaction was carried out in 10 μL for each sample, including 5 μL of SYBR Green Master Mix (Roche), 1 μL of cDNA template (1:25 template dilution) and 0.7 pmol/μL of each primer. The procedure used to obtain the RT-qPCR thermal profile was: 95 °C for 20 s, 40 cycles of 95 °C for 1 s and 60 °C for 20 s. The melting curve of each amplicon was revealed by the program from 60 to 95 °C, reading every 0.5 °C. The gene-specific amplification and the absence of primer-dimers were confirmed by the presence of single peaks for all genes. qPCR was carried out in triplicate (technical replicates) on cDNA, deriving from three independent cultures (biological replicates) and each assay included three no template negative controls for each primer pair.

### 4.5. Nitric Oxide (NO) Determination

NO levels were measured by monitoring the formation of nitrite, the oxidation product of NO, through the Griess assay [75]. At different times from dawn, diatom samples (50 mL) were collected by centrifugation at 2600 rcf for 15 min at 4 °C (Eppendorf 5810 R, Eppendorf AG, Hamburg, Germany). The pellets were washed in phosphate buffer (KH_2_PO_4_ 50 mM pH 7.5, 0.5 M NaCl) and centrifuged again under the same conditions. The final pellets were weighed, frozen in liquid nitrogen and kept at −80 °C until use. Samples were homogenized in 1 mL of phosphate buffer, sonicated two times at 30% amplitude for 1 min with a one-minute-break between the two sonication cycles. The samples were centrifuged at 13,000 rcf for 15 min at 4 °C and the supernatants were analyzed for nitrite content. Aliquots (300 µL) were incubated at room temperature (25 °C) with nitrate reductase (1 U/mL) and the enzyme cofactors: flavin adenine dinucleotide (100 µM) and nicotinamide adenine dinucleotide phosphate hydrogen (0.6 mM). After 2 h, samples were treated for 10 min in the dark with 300 µL of 1% (*w*/*v*) sulphanilamide in 5% H_3_PO_4_ and then with 300 µL of 0.1% (*w*/*v*) N-(1-naphthy)-ethylenediamine dihydrochloride for additional 10 min. The absorbance at 540 nm was measured in 1 mL glass cuvettes and the molar concentration of nitrite in the sample was calculated by interpolation from a standard curve generated using known concentrations of sodium nitrite (0–5 µM). Nitrite content in each sample was determined in triplicate (technical replicates) on samples deriving from three independent cultures (biological replicates).

### 4.6. Reactive Oxygen Species (ROS) Determination

ROS levels were measured in vivo using a fluorescent ROS-sensitive dye, 2′,7′-dichlorofluorescein diacetate (H_2_DCF-DA; Sigma-Aldrich, Saint Louis, MO, USA). At different times from dawn, diatom samples (15 mL) were incubated for 30 min in the dark with H_2_DCF-DA (20 µM final concentration). The cells were then collected by centrifugation, as described above, homogenized in phosphate buffer (0.5 mL), and sonicated as described above. The samples were centrifuged at 13,000 rcf for 15 min at 4 °C and the supernatants were analyzed for ROS content. Aliquots (5 µL) of samples were diluted in 100 µL of MilliQ water in a 96 multiwell plate and the fluorescence was measured using excitation and emission wavelengths of 485 and 530 nm, respectively. The molar concentration of ROS in the sample was calculated from a standard curve generated using known concentrations of 2′,7′-dichlorofluorescein (H_2_DCF; 0–1 µM). ROS content in each sample was determined in triplicate (technical replicates) on samples deriving from three independent cultures (biological replicates).

### 4.7. Thiols Determination

Three independent *S. marinoi* cultures were carried out under control (Sin150) and high sinusoidal light (Sin600) conditions. Cultures were collected at 6 h from dawn in both light conditions (i.e., at the maximal peak of light intensity) by centrifugation at 2600 rcf speed, 4 °C for 15 min (Eppendorf 5810 R, Eppendorf AG, Hamburg, Germany). Pellets were lyophilized using an Edwards lyophilizer and the dried pellets were weighed and kept at room temperature until analysis. Samples were finally analyzed by HPLC/LC–MS for ovothiol and glutathione determination, according to the protocol fully described in Milito et al., 2020 [23]. Briefly, thiols were extracted using a solution of acetonitrile:perchloric acid (1:1), reduced by adding DTT, and finally derivatized with 4-bromomethyl-7-methoxycoumarin (BMC). Samples were analyzed by reversed phase HPLC and thiol-BMC conjugates were detected with a diode-array detector at 330 nm. Isolated ovothiol-BMC adducts were characterized by High-resolution electrospray ionization mass spectrometry (HR-ESI-MS). Ovothiol B identification was confirmed by coelution with an authentic standard [23].

### 4.8. Data Analysis

Gene expression levels were normalized using the most stable reference genes in RT-qPCR using the sampling time 0 h (predawn) as the control condition for each experiment. Data were presented as mean ± standard error and statistical analyses were performed using the pairwise fixed reallocation randomization test by REST (*n* = 3 biological triplicate, *n* randomizations = 2000, relative expression software tool) [76]. Relative expression ratios above 2-fold and with *p* value ≤ 0.05 were considered significant. NO and ROS data were presented as mean ± standard deviation and analyzed by Kruskal–Wallis with a Dunn’s post hoc test (*n* = 3 biological triplicate). Kruskal–Wallis/Dunn’s test and linear correlation analyses (Pearson) were performed using PAST software package, version 3.14 [77]. Graphs were built using GraphPad Prism software V 6.01.

### 4.9. Nitric Oxide Synthase (Nos) Protein Sequence Analysis

The three *Homo sapiens* Nos protein sequences were downloaded from the NCBI protein database (iNOS-inducible: NP_000616.3, eNOS-endothelial: BAA05652.1 and nNOS-neuronal: NP_000611.1). The two *S. marinoi* (strain CCMP2092) Nos protein sequences were previously identified in *S. marinoi* [40], and were downloaded from the MMETSP website (Nos1 ID: MMETSP1039-20121108|3976_1; Nos2 ID: MMETSP1039-20121108|3419_1). The sequences were aligned using ClustalX and edited with GeneDoc software. The analysis of domains was performed using InterPro database.

## Figures and Tables

**Table 1:** Thiols determination in *S. marinoi.* The concentrations of ovothiol B and glutathione in *S. marinoi* under control (Sin150) and high sinusoidal (Sin600) light conditions are reported as mean ± standard deviation.

Light Condition	Ovothiol B Concentration	Glutathione Concentration
Low sinusoidal light (Sin150)	50 ± 10 µM	1.0 ± 0.3 mM
High sinusoidal light (Sin600)	110 ± 20 µM	2.3 ± 0.3 mM

**Table 2:** Pairwise Pearson correlation analyses conducted on all variables (fold gene expression ratios, NO and ROS levels). Different colors refer to different light conditions (red = high light (HL), blue = low light (LL) and green = dark (D)). HL, LL and D indicate the light intensity at which the indicated pair is positively correlated. * *p* < 0.05, ** *p* < 0.01 and *** *p* < 0.001 represent significance of positive correlation.

	NO	ROS	*ovoA*	*nos1*	*nos2*
**NO**		HL, LL	D	-	-
**EOS**	*** *p* < 0.001, *** *p* < 0.001		-	HL, D	-
***ovoA***	* *p* < 0.05	-		LL	HL, LL
***nos1***	-	* *p* < 0.05, * *p* < 0.05	** *p* < 0.01		LL
***nos2***	-	-	*** *p* < 0.001, *** *p* < 0.001	** *p* < 0.01	

## Supplementary Materials

The following are available online at https://www.mdpi.com/1660-3397/18/9/477/s1, Figure S1: Protein sequence alignment of *S. marinoi* Nos’s with human isoforms, Figure S2: *S. marinoi* growth curves, Table S1: *S. marinoi* cell growth rates and Table S2: Genes analyzed by RT-qPCR.
