# Supplementary material for: Insights into the Light Response of Skeletonema marinoi: Involvement of Ovothiol
Source: Mar Drugs. 2020 Sep 20;18(9):477. doi: 10.3390/md18090477 (PMC7551349; doi:10.3390/md18090477)
Supplement: Supplementary file 1 [file marinedrugs-18-00477-s001.pdf]

Article

Insights into the Light Response of *Skeletonema Marinoi*: Involvement of Ovothiol

Alfonsina Milito [1,2*], Ida Orefice [3], Arianna Smerilli [3], Immacolata Castellano [1], Alessandra Napolitano [4], Christophe Brunet [3] and Anna Palumbo [1,*]

1 Department of Biology and Evolution of Marine Organisms, Stazione Zoologica Anton Dohrn, Villa Comunale, 80121 Napoli, Italy; immacolata.castellano@szn.it

2 Current affiliation: Department of Molecular Genetics, Centre for Research in Agricultural Genomics, Cerdanyola, 08193 Barcelona, Spain

3 Department of Marine Biotechnology, Stazione Zoologica Anton Dohrn, Villa Comunale, 80121 Napoli, Italy; ida.orefice@szn.it (I.O.); arianna.smerilli@szn.it (A.S.); christophe.brunet@szn.it (C.B.)

4 Department of Chemical Sciences, University of Naples "Federico II", 80126 Naples, Italy; alessandra.napolitano@unina.it

* Correspondence: alfonsina.milito@szn.it, alfonsina.milito@cragenomica.es (A.M.); anna.palumbo@szn.it (A.P.); Tel.: +39-081-5833 (ext. 293/276)

----------------------------Supporting Information----------------------------

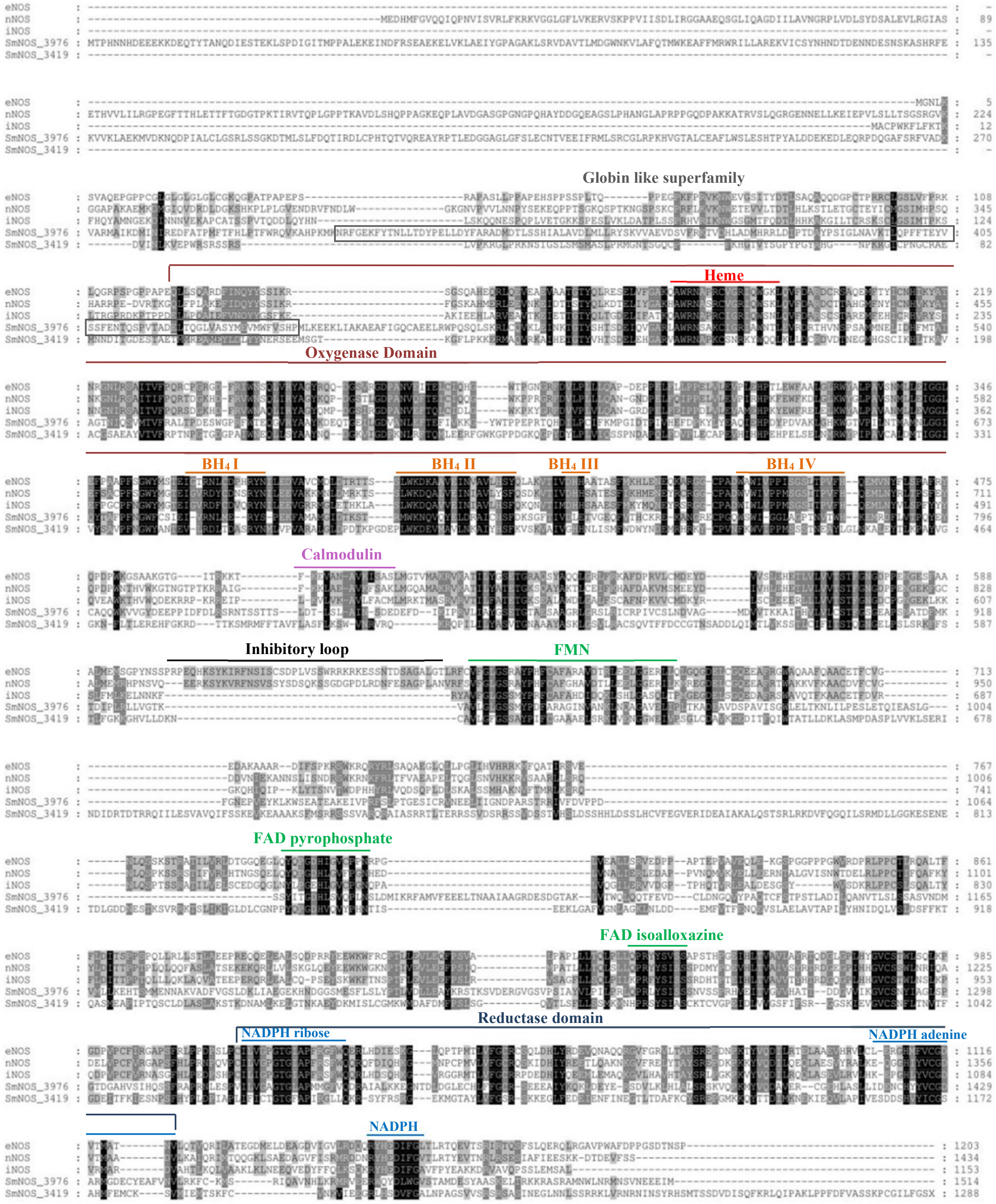

Figure S1. Protein sequence alignment of *S. marinoi* Nos's with human isoforms. The sequences of *Sm*Nos and human eNos, nNos, and iNos were aligned using ClustalX and Genedoc software. Black boxes indicate conserved residues in all five sequences, dark-gray boxes represent conserved residues in four sequences, and light-gray boxes represent conserved residues in three sequences. Amino acids that do not share similarity are unshaded. Putative cofactor binding sites for Heme, BH4, CaM, FMN, FAD pyrophosphate, FAD isoalloxazine, NADPH ribose, NADPH adenine, and C-terminal domain of NADPH are shown, as well as the inhibitory loop.

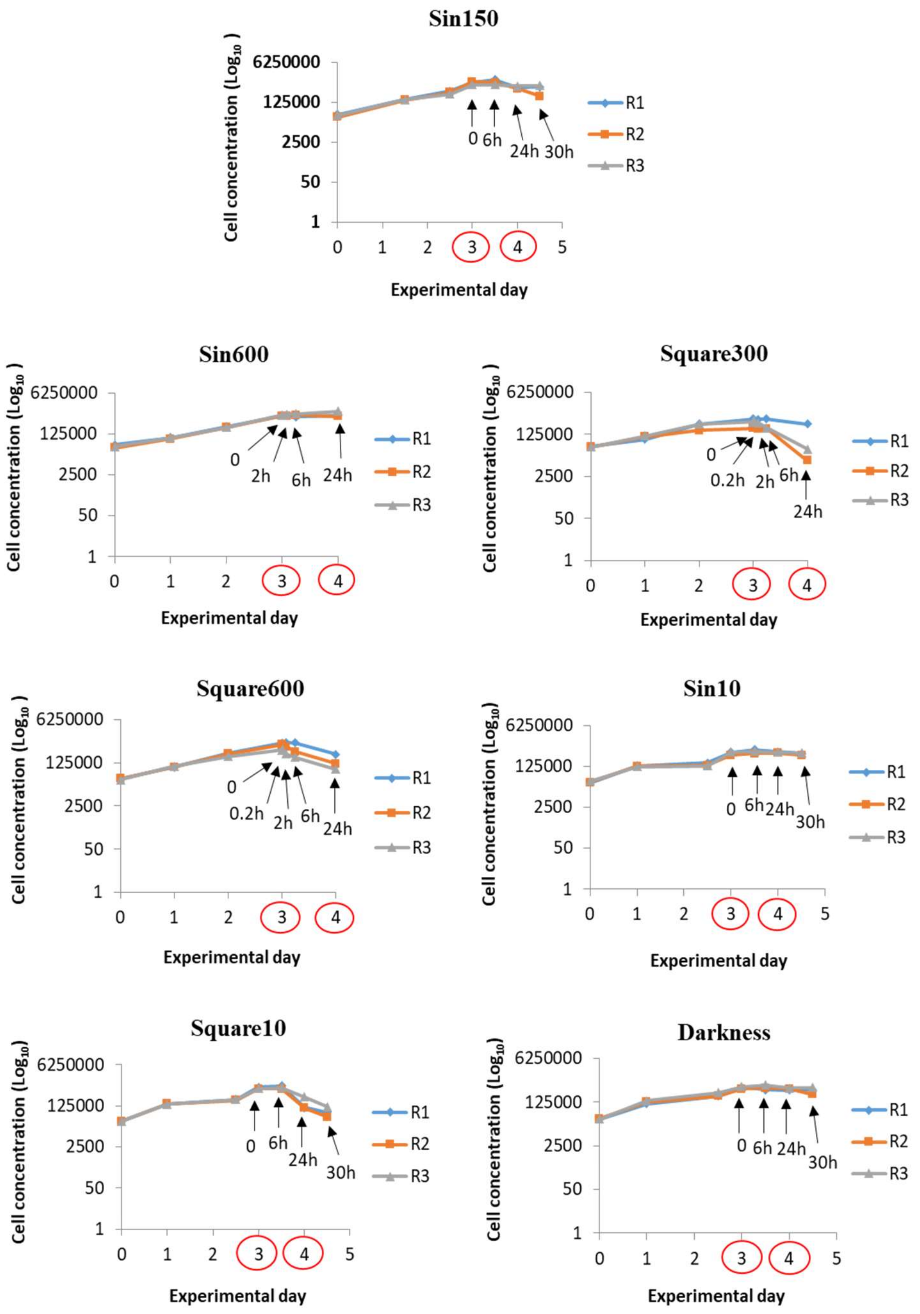

Figure S2. ***S. marinoi*** **growth curves.** Growth curves were obtained from three independent cultures (R1, R2, R3). Sampling days are highlighted by red circles and sampling times are reported for each experimental condition: low sinusoidal light with midday peak at 150 µmol photons s-1 m-2 (Sin150); high sinusoidal light with midday peak at 600 µmol photons s-1 m-2 (Sin600); high square-wave light with midday peak at 300 µmol photons s-1 m-2 (Square300); high square-wave light with midday peak at 600 µmol photons s-1 m-2 (Square600); very low sinusoidal light with midday peak at 10 µmol photons s-1 m-2 (Sin10); very low square-wave light with peak at 10 µmol photons s-1 m-2 (Square10); darkness.

Table S1. ***S. marinoi*** **cell growth rates.** Growth rates (μ, d^{-1}) at 0 and 24 h from light switch are indicated for each experimental condition as mean ± SD.

Light condition	0 h	24 h
Sin150	0.93 ± 0.07	- 0.49 ± 0.33
Sin600	1.12 ± 0.07	0.09 ± 0.21
Square300	0.34 ± 0.19	- 1.50 ± 1.40
Square600	0.78 ± 0.16	- 1.48 ± 0.42
Sin10	1.09 ± 0.16	0.09 ± 0.07
Square10	1.09 ± 0.05	- 1.53 ± 0.61
Dark	0.66 ± 0.13	- 0.14 ± 0.12

Table S2. Genes analyzed by RT-qPCR. Primer sequences, PCR amplicon sizes, temperature of annealing (Ta), primer efficiencies (E) and correlation factor (R^2) are reported.

Gene	Forward primer (5′⇒3′)	Reverse primer (5′⇒3′)	Amplicon size (bp)	Ta (°C)	E	R^2
ovoA	AAAGAGATGGCTCGCCTACA	GATTTGCAGCAGTCTCACCA	171	60	1.96	0.998
nos1	AAAGCCAGCCACAGATTCGA	ACTAAAGCCAAGACCAGCCC	228	60	1.97	0.989
nos2	TGGGTTTCGGTAGTTCTGCC	ACAACGTCGCTGTCCAGATT	159	60	1.97	0.998
